# Urban–rural differences in seasonal malaria chemoprevention coverage and characteristics of target populations in nine states of Nigeria: a comparative cross-sectional study

**DOI:** 10.1186/s12936-023-04825-7

**Published:** 2024-01-02

**Authors:** Taiwo Ibinaiye, Kunle Rotimi, Ayodeji Balogun, Adaeze Aidenagbon, Chibuzo Oguoma, Kevin Baker, Olabisi Ogunmola, Olusola Oresanya, Christian Rassi, Chuks Nnaji

**Affiliations:** 1 Malaria Consortium, 33 Pope John Paul Street, Maitama, Abuja-FCT, Nigeria; 2 Malaria Consortium, The Green House, 244–254 Cambridge Heath Road, London, E2 9DA UK; 3 Department of Global Public Health, Karolinska Institute, Stockholm, Sweden

**Keywords:** Malaria, Seasonal malaria chemoprevention, Rural -urban differences, Under 5, Caregivers

## Abstract

**Background:**

Differences between urban and rural contexts in terms of sociodemographic characteristics, geographical features and risk perceptions may lead to disparities in coverage and related outcomes of community-based preventive interventions, such as seasonal malaria chemoprevention (SMC). This study investigated urban–rural differences in SMC coverage and other programme outcomes, as well as child and caregiver characteristics of target populations in nine implementing states in Nigeria during the 2022 SMC round.

**Methods:**

This is a comparative cross-sectional study based on comprehensive end-of-round household surveys conducted in nine states where SMC was delivered in Nigeria in 2022. Data of 11,880 caregiver-child pairs were included in the analysis. Rural-urban differences in SMC outcomes and child and caregiver characteristics were assessed, first by using Pearsons’ chi-square test for independence for categorical variables. Univariate multilevel mixed-effect logistic regression models, with random intercepts for cluster units, were used to quantify the strength of association between location and each SMC coverage and related outcomes.

**Results:**

Significant urban-rural differences were observed in caregivers’ sociodemographic characteristics, such as age, gender, level of education, occupation status and health-seeking behaviour for febrile childhood illnesses. Disparities were also seen in terms of SMC coverage and related outcomes, with lower odds of the receipt of Day 1 dose direct observation of the administration of Day 1 dose by community distributors, receipt of the full three-day course of SMC medicines and receipt of SMC in all cycles of the annual round among children residing in urban areas, compared with those residing in rural areas. Similarly, urban-dwelling caregivers had lower odds of being knowledgeable of SMC and believing in the protective effect of SMC than rural-dwelling caregivers.

**Conclusion:**

Findings highlight observable urban-rural disparities in SMC programme delivery and related outcomes, as well as target population characteristics, underscoring the need for context-specific strategies to ensure optimal delivery of SMC and improve programme implementation outcomes in urban settings.

## Background

Malaria is a significant public health concern in Nigeria, with the country accounting for 38.4% of global malaria deaths in children aged under 5 years [1]. Having been endorsed by the World Health Organization (WHO) in 2012 for areas where malaria transmission is high and seasonal, accumulating evidence has shown that SMC using sulfadoxine-pyrimethamine and amodiaquine (SPAQ) is a highly effective strategy for preventing malaria in children under five years of age, who are most vulnerable to the disease [2, 3].

While malaria impact has historically been thought to be more in rural disease it is now recognized as an emerging threat in urban settings, particularly in rapidly urbanizing areas of sub-Saharan Africa [4, 5]. Given this threat, the delivery of population-level malaria prevention programmes in urban communities has received increasing attention [6, 7]. Following the success of SMC delivery in protecting at-risk children over the last decade, there have been recent efforts to expand its deployment and extend its benefits to new contexts, including urban settings. Consequently, SMC implementation contexts have expanded from traditionally rural settings, to being deployed in urban and peri-urban contexts, including the recent introduction of SMC in the metropolitan area of Abuja in Nigeria’s Federal Capital Territory (FCT) in 2022 as part of the city’s malaria prevention and control strategies [8].

Lessons from the delivery of SMC in urban settings indicate that geographical and socio-economic differences between urban and rural settings can have a significant impact on SMC campaign delivery [9]. For example, it was learnt that community engagement strategies suited for rural areas may be less suitable in urban target populations [10–14]. The complexity of the urban environment and less communal characteristics may also present operational challenges, such as the slower pace of door to-door delivery of SMC medicines during monthly campaigns in urban compared with rural settings, and difficulty in recruiting community distributors that are trusted by caregivers in urban areas [9].

Like other public health interventions, the effectiveness of SMC as a preventive strategy depends on optimal awareness and knowledge among communities where the intervention is implemented, as well as high coverage. However, differences between urban and rural contexts in terms of sociodemographic characteristics, geographical features, malaria risk perceptions and care seeking behaviour may lead to variations in SMC knowledge, perception and coverage [15, 16]. There is currently limited evidence on the nature and extent of such disparities in the context of SMC. Therefore, this study examined urban–rural differences in SMC coverage and other programme outcomes, as well as child and caregiver characteristics of target populations of eligible children in nine implementing states in Nigeria during the 2022 round.

## Methods

### Study design

This is a comparative cross-sectional analysis based on comprehensive end-of-round household surveys conducted in nine states where SMC was delivered in Nigeria in 2022.

### Study setting

**Fig. 1:**
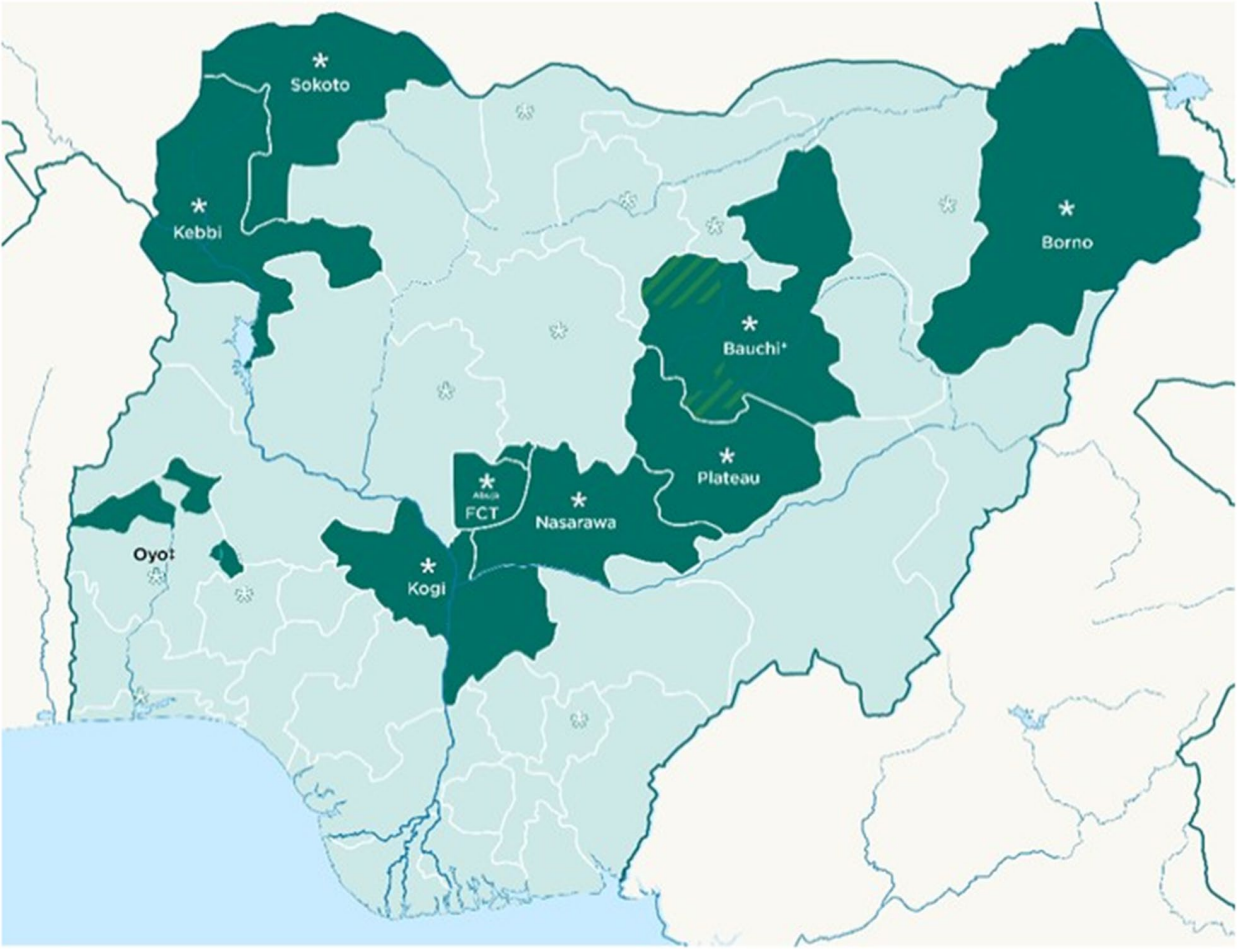
Map of Nigeria indicating the nine states represented in this study

This study used data from SMC campaigns implemented in Bauchi, Borno, Kebbi, Kogi, Nasarawa, Oyo, Plateau, Sokoto States, and the FCT in 2022 (Fig. 1). In 2022, SMC was implemented in all the LGAs across the listed states and FCT, except for Oyo where the intervention was implemented in only 6 LGAs (Fig. 1). SMC was introduced in the FCT, Oyo state and 12 LGAs in Kogi state that year, whereas the other states and LGAs in Kogi state had previous experience of implementing SMC. Five monthly cycles were implemented in the FCT, Kogi, Nasarawa, Oyo, Plateau and ten LGAs in Bauchi state, while four cycles were implemented in Borno, Kebbi, Sokoto and another ten LGAs in Bauchi state. The five-cycle SMC round was implemented from early June to early October 2022 whereas the four-cycle round was delivered from late June to late September 2022. Around 10.72 million SMC eligible children aged 3–59 months were targeted across the eight states and the FCT in 2022.

### Sampling and data collection process

The analysis required a minimum sample size of 390 caregiver-child pairs (195 urban and 195 rural dwellers) to be powered to 80%, at the 95% confidence level using a two-tailed test, to detect a statistically significant difference in SMC coverage between urban and rural settings. This assumed SMC coverage of 80% and 90% among children living in urban and rural communities, respectively based on previous findings from routine programme data. Surveys were carried out after the last monthly SMC cycle by independent evaluators. A multistage cluster sampling technique was used to select households with SMC-eligible children aged 3–59 months. Surveys were intended to achieve a representative sample of the target population of eligible children at country level and state levels. Sampling protocols aimed to achieve a self-weighted sample with sampling units selected with probability proportional to size. Only at the last stage of sampling (i.e. at the compound level) was a constant number of eligible children (one child per household) selected. Older children aged 5–10 years, if present in sampled households, were randomly selected to estimate the degree to which ineligible children received SMC as a measure of implementation quality.

Data were collected using structured questionnaires administered electronically via the SurveyCTO platform. Data on SMC coverage in eligible children (proportion of eligible children who received at least one dose of SMC medicines), receipt of the first dose under directly observed therapy (DOT), receipt of the full three-day course of SMC medicines and receipt of SMC in all monthly cycles. Surveys also enabled the collection of information on caregiver SMC awareness, knowledge and belief. Caregivers were also asked if they were visited by a lead mother in the past cycle. Lead mothers are trained community members who act as role models to other mothers in their communities, such as by reminding them to administer doses of SMC medicines on the second and third days following the first dose to achieve completion of the full three-day course. Where ineligible children are present in the household, data on their receipt of SMC medicines are also collected. Data on sociodemographic characteristics included child-level factors such as age, sex; while caregiver factors included age, gender, level of education, employment status and health-seeking behaviour for febrile childhood illnesses. Household-level factors included and place of residence (where rural or urban), mosquito net ownership, use of mosquito nets and indoor residual spraying.

### Statistical analysis

Descriptive statistics were used to explore the distribution of SMC outcomes and child and caregiver characteristics across rural and urban settings. Categorical variables were expressed as frequencies and percentages. Rural-urban differences in SMC outcomes and child and caregiver characteristics were assessed, first by using Pearsons’ chi-square test for independence for categorical variables. Two-level mixed-effect logistic regression models were used to quantify the strength of association between each of the pre-specified child- or caregiver-level SMC outcomes as dependent variables and household location (urban vs. rural) as independent variable (level 1), with random intercepts for cluster units (level 2). This analytical approach was taken given the clustered nature of survey data, as children and caregivers were sampled within household clusters (based on enumeration areas). The outcome variables of interest are the key SMC coverage and quality indicators described in the 2022 SMC implementation report [17]. Measures of association were presented as odds ratios (OR) with their corresponding 95% confidence intervals (CI), with statistical significance considered at p-value < 0.05. Forest plots of odds ratios were generated to visually illustrate the association between urban-rural residence and SMC outcomes.

## Results

The final analytic sample included data from 11,880 caregivers of eligible children from across the nine states without missing observations for any of the variables selected for analysis. Of these, 7,260 (61.1%) were rural dwellers while 4,620 (38.9%) resided in urban areas. There were no significant rural-urban differences observed in terms of child age and sex distributions. However, there were observable differences in caregivers’ sociodemographic characteristics, including age, gender, level of education, occupation status and health-seeking behaviour for febrile childhood illnesses. Urban caregivers were more likely to be female, older, literate, highly educated, without partners and engaged in engaged in sales/service/professional work (Table 1).

**Table 1:** Sociodemographic of children and their caregivers by location of residence

	URBAN		RURAL		Total		p-value
	n	%	n	%	n	%	
Child age							0.076
3-<12 m	332	7.2	542	7.5	874	7.4	
1-2years	1763	38.2	2621	36.1	4,384	36.9	
3-4years	2525	54.7	4097	56.4	6,622	55.7	
Child sex							0.590
Female	2293	49.6	3566	49.1	5,859	49.3	
Male	2327	50.4	3694	50.9	6,021	50.7	
Caregiver gender							< 0.001
Female	4018	87.0	6106	84.1	10,124	85.2	
Male	602	13.0	1154	15.9	1,756	14.8	
Caregiver age							< 0.001
Under 20 years	222	4.8	411	5.7	633	5.3	
20–29 years	1846	40.0	3111	42.9	4,957	41.7	
30–39 years	1792	38.8	2641	36.4	4,433	37.3	
40–49 years	548	11.9	839	11.6	1,387	11.7	
50–59 years	151	3.3	184	2.5	335	2.8	
60 or more years	61	1.3	74	1.0	135	1.1	
Caregiver marital status							0.001
Married/in a partnership	4284	92.7	6854	94.4	11,138	93.8	
Single/unpartnered	191	4.1	237	3.3	428	3.6	
Divorced/Widowed	145	3.1	169	2.3	314	2.6	
Caregiver literacy							< 0.001
No	1097	23.7	2500	34.4	3,597	30.3	
Yes	3523	76.3	4760	65.6	8,283	69.7	
Caregiver level of education							< 0.001
None	788	17.1	1833	25.2	2,621	22.1	
Informal or religious	845	18.3	1560	21.5	2,405	20.2	
Primary	654	14.2	1263	17.4	1,917	16.1	
Secondary	1646	35.6	2080	28.7	3,726	31.4	
Post-secondary	687	14.9	524	7.2	1,211	10.2	
Caregiver occupation							< 0.001
Unemployed	1300	28.1	2142	29.5	3,442	29.0	
Agriculture	674	14.6	2240	30.9	2,914	24.5	
Unskilled manual work	386	8.4	403	5.6	789	6.6	
Sales services and skilled manual	1979	42.8	2250	31.0	4,229	35.6	
Clerical, technical, professional or managerial	281	6.1	225	3.1	506	4.3	
Child use of mosquito net							< 0.001
No	322	10.0	284	5.6	606	7.3	
Yes	2885	90.0	4784	94.4	7,669	92.7	
Caregiver-reported fever							0.990
No	3270	70.8	5139	70.8	8,409	70.8	
Yes	1350	29.2	2121	29.2	3,471	29.2	
Malaria testing among febrile children							0.001
No	415	30.7	544	25.6	959	27.6	
Yes	935	69.3	1577	74.4	2,512	72.4	

### Urban–rural differences in SMC coverage, awareness, knowledge and perception

There were disparities in key SMC coverage and quality outcomes among children (Table 2). Compared with urban children, children in rural households had significantly better SMC outcomes in terms of receiving the first dose of SMC medicines (94.8% vs. 92.4%, p < 0.001), receiving the first dose under directly observed therapy (90.3% vs. 87.0%, p < 0.001), receiving the complete three-day course of SMC medicines (93.1% vs. 89.1%, p < 0.001) and receiving SMC medicines in all monthly cycles of the annual round (85.3% vs. 78.5%, p < 0.001). Similarly, notable urban-rural variations were observed in caregiver-level SMC outcomes, such as SMC awareness, knowledge, and perception, as well as access to peer-support through SMC lead mother visits, as summarized in Table 2.

**Table 2:** SMC outcomes of children and their caregiver by location of residence

	URBAN		RURAL		Total		p-value
	n	%	n	%	n	%	
Receipt of first dose of SPAQ on Day 1							< 0.001
Did not receive SMC drugs	351	7.6	380	5.2	731	6.2	
Received SMC drugs	4269	92.4	6880	94.8	11,149	93.8	
Receipt of first dose of SPAQ under directly observed therapy							< 0.001
No	554	13.0	665	9.7	1,219	10.9	
Yes	3715	87.0	6215	90.3	9,930	89.1	
Receipt of the full 3-day course of SPAQ							< 0.001
No	478	10.3	501	6.9	979	8.2	
Yes	4142	89.7	6759	93.1	10,901	91.8	
Receipt of SMC medicines in all cycle**s**							< 0.001
No	993	21.5	1067	14.7	2,060	17.3	
Yes	3627	78.5	6193	85.3	9,820	82.7	
Knowledge of SMC							< 0.001
Incomplete knowledge	2212	47.9	2941	40.5	5,153	43.4	
Complete knowledge	2408	52.1	4319	59.5	6,727	56.6	
Receipt of SMC medicines by age-ineligible children							0.014
No	852	74.0	1388	77.9	2,240	76.3	
Yes	300	26.0	394	22.1	694	23.7	
Lead mother visit							< 0.001
No	1281	30.0	1672	24.3	2,953	26.5	
Yes	2988	70.0	5208	75.7	8,196	73.5	
Caregiver belief in the protective effect of SMC							< 0.001
No	158	4.3	174	2.9	332	3.4	
Yes	3534	95.7	5924	97.1	9,458	96.6	

**Fig. 2:**
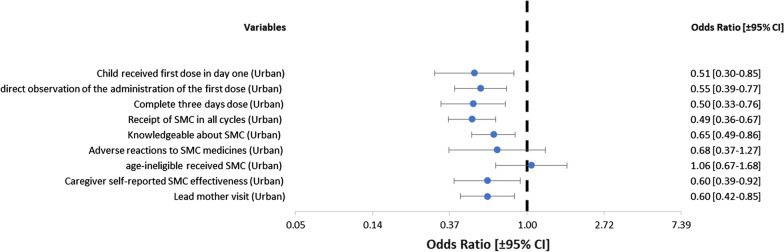
Forest plot of odds ratios indicating the association between urban-rural residence and SMC outcomes

Table 3, Fig. 2 present odds ratios of the association between urban-rural residence and SMC outcomes which were the quality indicators during SMC interventions. Compared with children residing in rural areas, those in urban settings had lower odds of receiving Day 1 SPAQ (OR: 0.507, 95% CI 0.304–0.846, p = 0.009), receiving Day 1 SPAQ under direct observation of community distributors (OR: 0.551, 95% CI 0.394–0.771, p < 0.001), receiving the full three-day course of SPAQ (OR: 0.500, 95% CI 0.330–0.757, p < 0.001) and receiving SMC in all cycles of the annual round (OR: 0.491, 95% CI 0.361–0.668, p < 0.001). Similarly, urban-dwelling caregivers had lower odds of being knowledgeable of SMC (OR: 0.649, 95% CI 0.491–0.859, p = 0.002), and believing in the protective effect of SMC (OR: 0.600, 95% CI 0.390–0.921, p = 0.020) than rural-dwelling caregivers. Compared with caregivers in rural areas, those living in urban areas had lower odds of being visited by lead mothers (OR: 0.600, 95% CI 0.423–0.852, p = 0.004).

**Table 3:** Results of univariate mixed-effects logistic regression models of association between rural/urban dwelling and seasonal malaria chemoprevention implementation outcomes in nine States in Nigeria (n = 11,880)

Variable	Category	Odds ratio	95% CI		p
Receipt of first dose of SPAQ on Day 1	Rural	Ref			
	Urban	0.507	0.304	0.846	0.009
Direct observation of the administration of the first dose of SPAQ	Rural	Ref			
	Urban	0.551	0.394	0.771	0.001
Receipt of full 3-day course of SPAQ	Rural	Ref			
	Urban	0.500	0.330	0.757	0.001
Receipt of SMC medicines in all cycles	Rural	Ref			
	Urban	0.491	0.361	0.668	< 0.001
Knowledge of SMC	Rural	Ref			
	Urban	0.649	0.491	0.859	0.002
Receipt of SMC by age-ineligible children	Rural	Ref			
	Urban	1.061	0.667	1.684	0.802
Caregiver belief in the protective effect of SMC	Rural	Ref			
	Urban	0.600	0.390	0.921	0.020
Lead mother visit	Rural	Ref			
	Urban	0.600	0.423	0.852	0.004

## Discussion

This analysis found considerable rural–urban disparities in child and caregiver characteristics across nine SMC-implementing states in Nigeria. It also highlights notable differences in SMC coverage and other implementation outcomes between rural and urban children and their caregivers. These findings have several implications for policy and practice, particularly for adapting the delivery of SMC and similar public health interventions to the contextual characteristics of implementation settings for optimal outcomes and impact.

Differences observed in terms of caregivers’ sociodemographic characteristics, such as age, gender, level of education, occupation status and health-seeking behaviour for febrile childhood illnesses are consistent with those reported by previous studies [17–22]. Unsurprisingly, urban caregivers were more likely to be literate, highly educated and engaged in engaged in sales/service/professional work. Despite these socioeconomic advantages, urban-dwelling caregivers had lower odds of SMC awareness, knowledge and belief than rural-dwelling caregivers. These seemingly paradoxical findings are consistent with those of previous studies in Nigeria and other African countries showing that rural respondents tended to have more positive attitudes and health-seeking practices than those living in urban settings despite the relatively higher literacy, level of education and broader socioeconomic status of urban dwellers [23, 24].

The success of public health interventions like SMC largely depends on caregivers’ knowledge and belief in the effectiveness of the intervention, which varied between rural and urban areas in this analysis. Hence, the lower odds of SMC awareness, knowledge and belief among urban caregivers may explain the lower coverage of SMC among children residing in urban areas, compared with those residing in rural areas. Gaps in urban caregiver’s knowledge and attitude towards SMC may reflect the complexity of urban living and how that shape health-seeking attitudes and practice. It may also reflect the inadequacy of SMC community engagement and delivery strategies in urban areas, as experiences have shown that typical SMC delivery strategies used in traditional, rural SMC settings may be unsuitable in urban implementation contexts [9–14]. As an example, the effectiveness of the social and behaviour change communication strategies that are highly effective in rural communities may be less so for urban populations.

Overall, these underscore the need for context-specific strategies to ensure optimal delivery of SMC and improve implementation outcomes in urban communities. There is thus a need to review, restrategize and tailor current SBCC strategies in urban SMC areas to navigate the contextual peculiarities of those settings. For example, the use of town announcers may be more effective in rural areas but may not produce desired result in urban areas where caregivers are more in tune with modern media outlets including social media platforms that may offer more access to information. Hence, for a more effective SMC intervention outcome there will be need to understand the unique characteristics of urban caregivers to develop suitable SBCC strategies during the SMC round.

Lower odds of direct observation of the administration of Day 1 SPAQ by community distributors may reflect the difficulty in recruiting SMC community distributors that are trusted by caregivers in urban areas as suggested by previous evidence [9]. This is also corroborated by the lower odds of being visited by lead mothers, who are community-based role models, among urban caregivers compared with rural caregivers. Moreover, urban settings in SMC implementation areas tend to have a significant proportion of households living in fenced homes or gated communities and estates, which may restrict access to community distributors and lead mothers and undermine their roles in the door-to-door SMC delivery model [25]. Moreover, community-based SMC delivery strategies may be better suited for more communal settings typical of rural communities, and less so for urban communities [26]. As such, it is imperative to tailor SMC campaign strategies in urban settings, which may include urban-specific adaptations of the roles of key implementing personnel like community distributors and lead mothers. Some of these strategies may include; the deployment of personnel with higher level of education as community distributors and lead mothers in urban setting; use of modern communication strategies like social media platforms during the interventions; use of fixed post distribution strategies instead of house-to-house models; use of private health facilities or outlets as supervising units instead of public health facilities among others.

### Strengths and limitations

Strengths of this analysis include its use of independent surveys conducted by external investigators not affiliated with SMC programmes, its large analytic sample, and its inclusion of nine states allowing generalizability of its results. Its limitations include reliance on self-reporting by caregivers, particularly for variables such as caregiver literacy which may have been subject to social desirability bias. Also, the length of time since the introduction of SMC in these locations was not considered in the analysis. This may have influenced SMC delivery outcomes, especially as SMC was first introduced in mostly rural areas with more recent introductions in urban contexts. Further research could explore the extent of the influence of such differences.

## Conclusions

This study highlights the urban-rural differences in child, caregiver and household characteristics and SMC outcomes in nine states in Nigeria. Findings have several implications for adapting and contextualizing the delivery of SMC and similar public health interventions, underscoring the need for context-specific strategies to ensure optimal delivery and impact.

## Data Availability

Data employed in this study are available from the authors upon reasonable request.

## Abbreviations

CI: Confidence interval
DOTs: Directly observed therapy
FCT: Federal capital territory
KOICA: Korea International Cooperation Agency
LGA: Local Government Area
NHREC: National Health Research Ethics Committee
OR: Odd Ratio
SBCC: Social and Behavior Change Communication
SMC: Seasonal malaria chemoprevention
SPAQ: Sulfadoxine/Pyrimethamine + Amodiaquine
WHO: World Health Organization
